# Mechanism of the Anti-inflammatory Effect of Curcumin: PPAR-γ Activation

**DOI:** 10.1155/2007/89369

**Published:** 2008-01-17

**Authors:** Asha Jacob, Rongqian Wu, Mian Zhou, Ping Wang

**Affiliations:** ^1^Division of Surgical Research, North Shore University Hospital and Long Island Jewish Medical Center, 350 Community Drive, Manhasset, NY 11030, USA; ^2^Center for Immunology and Inflammation, The Feinstein Institute for Medical Research, 350 Community Drive, Manhasset, NY 11030, USA

## Abstract

Curcumin, the phytochemical component in turmeric, is used as a dietary spice and a topical ointment for the treatment of inflammation in India for centuries. Curcumin (diferuloylmethane) is relatively insoluble in water, but dissolves in acetone, dimethylsulphoxide, and ethanol. Commercial grade curcumin contains 10–20% curcuminoids, desmethoxycurcumin, and bisdesmethoxycurcumin and they are as effective as pure curcumin. Based on a number of clinical studies in carcinogenesis, a daily oral dose of 3.6 g curcumin has been efficacious for colorectal cancer and advocates its advancement into Phase II clinical studies. In addition to the anticancer effects, curcumin has been effective against a variety of disease conditions in both in vitro and in vivo preclinical studies. The present review highlights the importance of curcumin as an anti-inflammatory agent and suggests that the beneficial effect of curcumin is mediated by the upregulation of peroxisome proliferator-activated receptor-γ (PPAR-γ) activation.

## 1. INTRODUCTION

Turmeric, *Curcuma longa* Linn., plant is a perennial herb belonging to the ginger family, *Zingiberaceae*, and is generally cultivated in south and southeast tropical Asia. The rhizome, which is also referred to as the root of this plant, is the most useful part and is used as a dietary spice for centuries. It has been used both orally and as a topical ointment to treat a variety of disorders. It is widely used in traditional Indian Ayurvedic medicine to treat hepatic disorders, anorexia, cough, diabetic wounds, rheumatoid arthritis, and sinusitis. Turmeric paste in slaked lime is a popular home remedy for the treatment of inflammation and wounds. Ancient texts of Indian medicine describe the use of curcumin in inflammatory diseases, wound healing, and abdominal problems [1].

## 2. CHEMICAL PROPERTIES

Curcumin, the most active component of turmeric, makes up 2–5% of this spice. The yellow color of the turmeric is due to the curcumin compound. Curcumin (C_21_H_20_O_6_) was first described in 1910 by Lampe and Milobedeska and shown to be a diferuloylmethane, 1,7-bis (4-hydroxy-3-methoxyphenyl)-1,6-heptadiene-3,5-dione [2], and is practically insoluble in water. Curcumin is a bis-*α*-*β*-unsaturated *β*-diketone; under acidic and neutral conditions, the bis-keto form of the compound predominates, and at pH above 8, the enolate form is generally found [3]. Hence at pH 3–7, it acts as an extraordinarily potent H-atom donor and above pH 8, it acts mainly as an electron donor, a mechanism more suitable to the scavenging or antioxidant properties of curcumin [4]. Curcumin is quite unstable at basic pH and degrades within 30 minutes. Human blood or antioxidants such as ascorbic acid, or the presence of 10% fetal bovine serum in the culture media prevents this degradation [5]. Curcumin has a molecular weight of 368.7 and the commercial grade curcumin contains curcuminoids, 10–20% desmethoxycurcumin and less than 5% bisdesmethoxycurcumin [3]. The commercial grade curcumin is just as effective as pure curcumin in preclinical models of carcinogenesis [6].

## 3. PHARMACOKINETIC AND PHARMACODYNAMIC PROPERTIES

Absorption, metabolism and tissue distribution are important parameters to render a compound to be used as a therapeutic agent. In this regard, in an early study where curcumin was administered to rats at a dose of 1 g/kg body weight in diet, 75% of the dose was excreted in the feces and trace amounts were present in urine [7]. A few years later, it was determined that 60% of orally administered curcumin was absorbed but most of it has been changed to glucuronide and sulphate conjugates and excreted in the urine [8]. Intravenous or intraperitoneal administration of curcumin in rodents resulted in the presence of large quantities of curcumin and its metabolites in the bile. This suggests that curcumin undergoes transformation during absorption via the intestine and possibly recirculates [9, 10]. Another study showed that coadministration of curcumin with piperine, a compound found in pepper vine and peppers, increased the bioavailability of curcumin following oral dosing presumably due to the inhibition of xenobiotic glucuronidation by piperine [11]. Thus, curcumin exhibits low systemic bioavailability after oral dosing in rodents and may undergo intestinal metabolism.

Only a limited number of studies have been shown for the pharmacokinetic properties of curcumin in humans and majority of these studies were conducted in cancer patients. In this regard, Cheng et al. administered 0.5 to 8 g daily of curcumin orally for 3 months to patients with high-risk premalignant conditions of the bladder, skin, cervix, stomach, or oral mucosa. Serum concentrations of curcumin peaked at 1-2 hour(s) posttreatment with a gradual decline within 12 hours. The 8 g/day resulted in about 1.75 *μ*M curcumin in the serum [12]. In a pilot study using a standardized oral *Curcuma* extract, doses up to 180 mg of curcumin per day were administered to patients with advanced colorectal cancer for up to 4 months without much toxicity [13]. In another Phase I study of 15 patients with advanced colorectal cancer, six patients were given 3.6 g of curcumin daily for up to 4 months. This daily dosing resulted in the detectable level of curcumin and its conjugates in plasma. Urine samples from these patients showed 0.1 to 1.3 *μ*M curcumin and trace levels of its conjugates. Since curcumin can be detected in the urine samples, urine analysis could be used as the measure of general compliance and the assessment of inter- and intraindividual variability [14].

Along with the dose studies, malignant colorectal tissues were analyzed in patients consuming 3.6 g of curcumin daily for 7 days prior to surgery. The concentrations of curcumin in normal and malignant colorectal tissues of patients were 12.7±5.7 and 7.7±1.8 nmol/g tissue, respectively. Curcumin conjugates were seen in the intestinal tissue of these patients and trace levels of curcumin were found in peripheral circulation [14]. Thus, a daily oral dose of 3.6 g of curcumin resulted in a pharmacologically efficacious level in colorectal tissues. Further studies are warranted for the efficacy of this compound in other diseases as well as different types of cancer.

## 4. BENEFICIAL EFFECTS

After a long-term use in traditional Ayruvedic medicine, modern scientific community discovered that curcumin has beneficial effects on a variety of diseases and pathological conditions [15]. Curcumin has shown to possess anticancer effects by blocking transformation, tumor initiation, tumor promotion, invasion, angiogenesis, and metastasis [2]. It has been demonstrated to have a dose-dependent chemopreventive effect in animal systems of colon, duodenal, stomach, esophageal, and oral carcinogenesis [16]. Low incidence of colon cancer among Indians has been attributed to the use of turmeric in Indian cooking [17]. In addition to its anticancer effects, curcumin has been effective against a variety of conditions in in vitro and in vivo preclinical studies [15]. Curcumin has shown to be effective against atherosclerosis and myocardial infarction. The administration of curcumin reduced blood sugar and glycosylated hemoglobin levels in an alloxan-induced rat model of type 2 diabetes. Curcumin appears to suppress oxidative damage, inflammation, cognitive deficits, and amyloid accumulation in Alzheimer's disease. In addition, curcumin appears to show protective effects in cystic fibrosis, human immunodeficiency virus, and experimental alcoholic liver disease.

Treatment with curcumin in an animal model of wound healing produced large infiltration of macrophages, neutrophils, and fibroblast compared to untreated wounds. The treatment resulted in enhanced expression of fibronectin and collagen by fibroblasts and increased the rate of formation of granulation tissue suggesting an enhancement in wound healing [18]. Curcumin is shown to modulate angiogenesis and uncontrolled angiogenesis had been associated with pathological conditions such as tumor growth and metastasis, rheumatoid arthritis, diabetic retinopathy, and hemangiomas [19, 20]. Curcumin treatment results in inhibition of angiogenic differentiation of human umbilical vein endothelial cells (HUVEC) [21], and inhibits basic fibroblast growth factor-induced corneal neovascularization in the mouse cornea [22], indicating its antiangiogenic effect. Curcumin is also a strong antioxidant compared to vitamins C and E [23]. Oxidative stress plays a major role in the pathogenesis of various diseases including myocardial ischemia, cerebral ischemia-reperfusion injury, hemorrhage, shock, hypoxia, and cancer. In a hemorrhage/resuscitation injury model, pretreatment with curcumin resulted in a significant decrease in liver enzyme, aspartate transaminase, and the liver cytokines, IL-1*α*, IL-1*β*, IL-2, IL-6, and IL-10 [24].

Perhaps the most important effect of curcumin is its anti-inflammatory properties and this is the major focus of this review. Only a few clinical studies have been reported on the effect of administration of curcumin on inflammatory diseases [25–28]. However, curcumin has been known to possess anti-inflammatory activity in experimental animals [29]. In this regard, we have recently shown that curcumin has beneficial effects in sepsis [30]. Male Sprague Dawley rats were treated with a bolus intravenous injection of 0.2 *μ*mol of curcumin followed by a continuous infusion of 0.24 *μ*mol/day for 3 days via a 2-mL alzet pump. Then the rats were subjected to sepsis by cecal ligation and puncture (CLP), a widely used animal model of sepsis. Twenty hours following CLP (i.e., the late stage of sepsis), the rats were killed and the blood and tissue samples were collected. The blood samples were analyzed for tissue injury parameters, alanine aminotransferase, aspartate aminotransferase, lactate, and TNF-*α*. As expected, sepsis induced a two-to-three-fold increase in the circulating levels of these injury markers compared to sham controls. Pretreatment with curcumin significantly reduced these levels to that of sham. Similar results were observed when curcumin was administered 5 hours after the onset of sepsis. In an additional group of animals, a 10-day survival study was conducted after CLP in animals pretreated with curcumin for 3 days. Sepsis caused a 56–69% mortality rate while pretreatment with curcumin improved the survival rate to 100% throughout the 10-day observation period. Thus, we have demonstrated the anti-inflammatory effect of curcumin in an in vivo experimental model of sepsis. We have also shown that pretreatment with 50 *μ*M curcumin in a macrophage cell line, RAW 264.7 cells, produced 23% and 71% reduction in LPS-induced increases in TNF-*α* gene expression and protein levels, respectively [30]. At 100 *μ*M curcumin, reduction by 60% and 99% in the LPS-stimulated increasse in TNF-*α* gene expression and protein levels were observed, respectively. These data prompted us to explore the potential mechanisms associated with curcumin-induced anti-inflammatory effects.

## 5. POTENTIAL MECHANISMS

The mechanism by which curcumin induces its anti-inflammatory effects is yet to be elucidated. Studies have shown that peroxisome proliferator-activated receptor gamma (PPAR-*γ*) has been associated with anti-inflammatory effects. PPARs belong to the superfamily of nuclear receptors consisting of three genes that give rise to three different subtypes, PPAR-*α*, PPAR-*δ*, and PPAR-*γ*. Among them, PPAR-*γ* is the most widely studied form. Upon ligand binding, PPAR-*γ* forms heterodimers with the retinoid X receptor and binds to a peroxisome proliferation response element (PPRE) in a gene promoter leading to regulation of gene transcription [31]. In that regard, we have recently shown that gene and protein levels of PPAR-*γ* in the liver decreased by approximately 50% at 20 hours after the onset of sepsis. Pretreatment with curcumin for 3 days at 0.24 *μ*mol/kg body weight in these septic rats produced 45% and 65% increase in PPAR-*γ* mRNA and protein levels, respectively. The mRNA and protein levels of PPAR-*γ* in the treatment group were similar to sham controls [30]. To confirm that the beneficial effect of curcumin in sepsis is mediated through PPAR-*γ* pathway, a separate group of animals were treated for 3 days with PPAR-*γ* antagonist, GW9662, at 1.5 mg/kg along with curcumin at 0.24 *μ*mol/kg body weight. Then, rats were subjected to sepsis by CLP and 20 hours after surgery, blood and tissue samples were collected. Concurrent administration of curcumin and GW9662 in the septic rats completely abolished the effects of curcumin on serum levels of the liver enzymes, ALT and AST, lactate, and TNF-*α* [30]. Furthermore, in vitro using RAW 264.7 cells, pretreatment with 50 and 100 *μ*M curcumin increased PPAR-*γ* mRNA levels by 86% and 125%, respectively, compared to LPS treatment alone. Consistent with this, immunohistochemical staining of RAW 264.7 cells with PPAR-*γ* antibody showed increased nuclear PPAR-*γ* staining in cells pretreated with 100 *μ*M curcumin compared to LPS alone. This suggests that the beneficial effect of curcumin appears to be mediated by the upregulation of PPAR-*γ* [30].

Both in vivo and in vitro studies have shown that activation of PPAR-*γ* by thiazolidinediones (TZDs), the class of insulin-sensitizing drugs, or 15d-PG-J_2_ has anti-inflammatory effects [32–34]. TZDs are the synthetic agonists of PPAR-*γ* and PGJ_2_ series have been identified as the natural ligand of PPAR-*γ*. In that regard, Zingarelli et al. showed that PPAR-*γ* expression was markedly reduced in lung and thoracic aorta after CLP sepsis. Furthermore, in vivo treatment with 15d-PGJ_2_ or ciglitazone, one of the TZDs, following CLP ameliorated hypotension and survival, blunted cytokine production and reduced neutrophil infiltration in lung, colon, and liver. These beneficial effects of PPAR-*γ* ligands were associated with the reduction of I*κ*B kinase complex, JNK activation, and reduction of NF-*κ*B and AP-1 pathways [32]. Recent evidence suggests that PPAR-*γ* ligands exert their effects in HT-29 colon cancer cells by phosphorylation of the PPAR-*γ* by the extracellular signal-regulated kinase 1/2, thereby causing a physical interaction with the p65 subunit of the NF-*κ*B preventing the activation of the NF-*κ*B pathway [35]. The inhibition of cell signaling pathways, Akt, NF-*κ*B, AP-1, or JNK, has been implicated as the mechanism responsible for apoptosis induction by curcumin. A recent study reported that curcumin potentiates the antitumor effect of gemcitabine in pancreatic cancer by suppressing proliferation, angiogenesis, and downregulating NF-*κ*B and NF-*κ*B-regulated gene products [36]. However, it is plausible that curcumin induced anti-inflammatory effect caused by the upregulation of PPAR-*γ* is associated with the NF-*κ*B pathway. Future studies are warranted for such conclusion.

Numerous studies have shown the importance of curcumin as a potent immunomodulatory agent in T cells, B cells, neutrophils, natural killer cells, dendritic cells, and macrophages [37]. In that regard, we have shown that curcumin induces apoptosis in human neutrophils [38]. Neutrophils are the first line of host immune defense against foreign substances and their biological activities are tightly regulated by apoptosis. Delayed neutrophil apoptosis has been associated with acute lung injury and sepsis [39–41]. We first examined the effect of curcumin on both spontaneous neutrophil apoptosis and apoptosis of neutrophils following transmigration across a human lung endothelium-epithelium bilayer. The results showed that curcumin increased constitutive neutrophil apoptosis and abrogated the transbilayer migration-induced delay in neutrophil apoptosis. To determine the impact of curcumin on neutrophil function, we performed myeloperoxidase activity and migration assays. Curcumin treatment decreased neutrophil migration and myeloperoxidase release indicating a reduction in neutrophil activation. To elucidate the potential mechanism, we have examined the effect of curcumin on p38 mitogen-activated protein kinase and caspase-3 activity. A marked increase in p38 phosphorylation and caspase-3 activity was observed in the presence of curcumin. Treatment of p38-specific inhibitor, SB203580, suppressed both curcumin-induced apoptosis and caspase-3 activation. From this study, we concluded that curcumin induces apoptosis in human neutrophil and its effect is mediated by the activation of p38 and caspase-3 activity.

## 6. FUTURE STUDIES AND PERSPECTIVES

In this review, we highlighted the importance of curcumin as an anti-inflammatory compound. We also demonstrated that the beneficial effect of curcumin in sepsis appears to be mediated by the upregulation of PPAR-*γ*, leading to the suppression of proinflammatory cytokine, TNF-*α* expression and release. Studies on the effect of both synthetic and natural ligands of PPAR-*γ* on polymicrobial sepsis are also reviewed. Curcumin effect on apoptosis and potential mechanisms isalso discussed.

In the future, we will explore the mechanism by which curcumin-induced upregulation of PPAR-*γ* leads to the suppression of TNF-*α* release to provide protection in sepsis. The major question we need to address is whether curcumin-induced anti-inflammatory effects are direct or indirect. One possible scenario is that curcumin as a ligand binds to the PPAR-*γ* receptor and heterodimerizes with the retinoic acid receptor and directly binds to the PPRE of TNF-*α* gene itself or genes which codes for its upstream mediators and inactivates them. Secondly, curcumin binds to its own receptor and the ligand-receptor interaction activates signaling pathways leading to the upregulation of PPAR-*γ* and the subsequent suppression of inflammatory cytokine release. In vitro studies with macrophages are recommended to dissect the precise mechanism of curcumin-induced anti-inflammatory effects. Another question we need to address is whether curcumin itself or its metabolites is the active compound responsible for anti-inflammatory effects. Prior studies on the anti-inflammatory nature of curcumin were done on curcumin itself. Further studies with curcumin metabolites, curcumin sulfate, and curcumin glucuronide will warrant answering such questions. Several studies suggest that PPAR-*γ* ligand, 15d-PGJ_2_, protects organs from tissue injury caused by ischemia/reperfusion injury, hemorrhage/resuscitation, or endotoxemia [42–44]. The effect of curcumin on other models of tissue injury should also be explored. Thus, more in vitro and preclinical researches are needed to render curcumin or its metabolites as therapy for sepsis and/or other models of tissue injury.
